# Expression of Gp78/Autocrine Motility Factor Receptor and Endocytosis of Autocrine Motility Factor in Human Thyroid Cancer Cells

**DOI:** 10.7759/cureus.4928

**Published:** 2019-06-17

**Authors:** Sam M Wiseman, Liliana D Kojic, Katayoon Kassian, Steven J Jones, Bharat Joshi, Ivan R Nabi

**Affiliations:** 1 Surgery, St. Paul’s Hospital & University of British Columbia, Vancouver, CAN; 2 Cellular & Physiological Sciences, University of British Columbia, Vancouver, CAN; 3 Bioinformatics, British Colombia / BC Cancer Agency – Vancouver Centre, Vancouver, CAN; 4 Genome Sciences Centre, British Columbia / BC Cancer Agency – Vancouver Centre, Vancouver, CAN

**Keywords:** gp78 e3 ubiquitin ligase, autocrine motility factor, endocytosis, thyroid cancer

## Abstract

Gp78/autocrine motility factor receptor (Gp78/AMFR) is a cancer-associated endoplasmic reticulum-localized E3 ubiquitin ligase and also the cell surface receptor for autocrine motility factor (AMF). The study objective was to determine the association between Gp78/AMFR and AMF endocytosis in thyroid cancer cells. Gp78/AMFR expression and AMF internalization were measured in differentiated thyroid cancer (DTC) and anaplastic thyroid cancer (ATC) cell lines and in freshly resected human papillary thyroid cancers (PTC) relative to benign thyroid tissue. Spheroid-like aggregates generated from explants of cancer, goiter, and collateral thyroid tissue were assessed for expression of cancer stem cell markers, surface Gp78/AMFR and AMF endocytosis. DTC cell lines showed elevated total and surface Gp78/AMFR and AMF internalization relative to ATC lines. Gp78/AMFR, Oct-4 and Sox-2 protein expression, Gp78/AMFR surface expression and AMF internalization were elevated in PTC-derived aggregates relative to fibroblasts. Elevated levels of Gp78/AMFR expression and AMF internalization in PTC were associated with expression of cancer stem cell markers. Gp78/AMFR expression and AMF uptake are more closely associated with DTC compared to benign thyroid lesions or ATC and with PTC-derived cancer stem-like cells.

## Introduction

Thyroid nodules are extremely common in the general population, being palpable in 5% of people and diagnosed by high resolution ultrasound in greater than half of people. Thyroid cancer tends to present as a thyroid nodule and, fortunately, only a small minority of thyroid nodules are eventually diagnosed as being malignant [1]. The steady rise in thyroid cancer incidence, which has been observed for greater than 20 years, has largely been attributed to increased utilization of diagnostic imaging [1]. Thyroid cancers, evaluated in [2], included papillary carcinoma (PTC) (80-85%) and follicular carcinoma (FTC) (10-15%) that are generally grouped as differentiated thyroid cancer (DTC), as well as highly aggressive anaplastic thyroid cancer (ATC) (1-2%) believed to arise from pre-existing DTC. The vast majority of DTC patients who undergo thyroid cancer treatment have an excellent prognosis. However, 5% of DTC patients develop progressive radioactive iodine-resistant metastatic disease and have very limited treatment options. The five-year disease-specific survival of these DTC patients is less than 50% [3] and fewer than 15% of these individuals survive past 10 years [4]. However, distinguishing DTC from benign thyroid lesions has remained a challenge. Simulation model analysis shows that accurately distinguishing benign from malignant thyroid nodules would lead to a reduction in the number of diagnostic thyroid operations, operative morbidity and cost [5]. Unlike DTC, ATC represents one of the most fatal human cancer types, and individuals diagnosed with ATC have few treatment options [2]. Elevated expression of autocrine motility factor (AMF) and its receptor, Gp78/autocrine motility factor receptor (Gp78/AMFR), promotes tumor cell motility and metastasis and is closely associated with malignancy in several different cancer types [6]. In human cancers, Gp78/AMFR expression correlates with aggressive cancer biology, and a worsened outcome for lung, tongue, esophagus, stomach, colon, rectum, liver, breast, thymus and skin cancers [6]. Notably, in bladder, colorectal, gastric, skin and esophageal cancers, Gp78/AMFR is either absent, or expressed at significantly lower levels in adjacent normal tissue [6]. Gp78 is located throughout the endoplasmic reticulum (ER) of cells. The anti-Gp78 monoclonal antibody, 3F3A, has extensively been utilized to study Gp78/AMFR in cancer, and more recently has been shown to selectively recognize a non-phosphorylated (on Serine 538) form of Gp78 [6, 7]. Interestingly, a Gp78/AMFR knockout mouse develops spontaneous liver and colon cancers, suggesting that Gp78/AMFR may have a tumor suppressor role in these cancer types [8]. Recent tissue microarray analysis (TMA) of separate cohorts of colon and rectal cancers showed unexpectedly that Gp78/AMFR expression was associated with improved patient survival in colon cancer, but with a worse prognosis in rectal cancer [9]. Together these observations suggest both tumor suppressor and promoter functions for Gp78.

Gp78/AMFR internalization of AMF, via a distinct raft-dependent endocytic pathway to the ER, is increased in metastatic cancer cells [10-11]. AMF overexpression induces cell transformation, tumorigenicity, and metastasis [12]. In this study our objective was to determine the association between Gp78/AMFR and AMF endocytosis with thyroid cancer.

## Materials and methods

Antibodies and reagents 

Monoclonal rat IgM antibody against Gp78/AMFR (3F3A) was used in the form of ascites fluid [13]. Alexa-488 and Alexa-647 conjugated anti-rat secondary antibodies were purchased from Molecular Probes (Eugene, OR). Anti-Oct-4 rabbit polyclonal (ab18976) and anti-Sox2 monoclonal (ab75485) antibodies were from Abcam (San Francisco, CA). Rabbit PGI (Type XI), propidium iodide (PI), collagenase-1, hyluronidase and pronase were purchased from Sigma (St. Louis, MO). AMF was conjugated to fluorescein isothiocyanate (FITC) with the Fluorescein-EX protein labeling kit (Molecular Probes).

Cell lines

Two papillary (TCP1 and KTC1), one follicular (FTC133), and three anaplastic (ACT1, T235, T238) human thyroid cancer cell lines were a kind gift from Dr. Schweppe (University of Colorado, Denver, CO). Cells were maintained in complete RPMI 1640 containing 10% fetal bovine serum (FBS). To minimize phenotypic drift, all cell lines were passaged two to three times after recovery from frozen stocks before initiating the experiments and maintained in culture for a maximum of 10-12 passages.

Human thyroid tissue microarrays

Human tissue microarrays were constructed as previously described [14]. The TMA included 100 benign thyroid lesions (54 goiters, 10 Hurthle cell adenomas, four hyperplastic nodules, three cases of Hashimoto’s thyroiditis, and three cases of lymphocytic thyroiditis), 90 PTCs, six FTCs, and an additional 32 ATC cases. Thus, thyroid tumors from 228 patients were evaluated on the TMA. The TMA also included lymph node specimens from 23 of the PTC cases. The clinical and pathological characteristics of the TMA thyroid tumors were previously described [14].

Gp78/AMFR expression was evaluated by immunohistochemistry. Briefly, TMA blocks were cut into 4-um sections using a Leica microtome (Leica Microsystems Inc., Richmond Hill, Ontario, Canada) and the sections transferred to adhesive-coated slides for immunohistochemical staining. Anti-Gp78/AMFR (3F3A) monoclonal antibody dilutions were validated using cores prepared from thyroid cancer cell pellets (TCP-1, KTC-1, FTC-133) to determine the optimal Gp78/AMFR dilution, and compared to the expression determined by flow cytometry and Western blotting [11]. The TMA sections stained with anti-Gp78/AMFR antibody (3F3A) were scored by two pathologists blinded to all clinical data. Any inter-pathologist disagreement was resolved by a third pathologist. Gp78/AMFR expression levels were given binary designations, in which Gp78/AMFR staining was regarded as positive or over-expressed, when greater than 10% of the cells examined showed cytoplasmic staining. The scoring system used the most clinically relevant and reproducible cutpoint for this marker. All scores were recorded by the pathologists on computer into a standardized TMA case map that corresponded to each TMA section (Microsoft Excel; Microsoft, Redmond, WA) and all data were processed by custom TMA-deconvoluter software (developed using the Perl programming language). The deconvoluted data then were manually transferred into a master database (Microsoft Excel; Microsoft, Redmond, WA), which also included all collected clinical and pathologic data for statistical analysis.

Human thyroid tumour dissociation and labeling

Surgical specimens of human thyroid tumors were obtained from the practice of Dr. Sam M. Wiseman, a thyroid surgeon based at St. Paul’s Hospital, Vancouver, British Columbia, Canada. This study was carried out with approval of the Research Ethics Board of the Providence Health Care Research Institute at the University of British Columbia (Ref No. H06-00220). Thyroid specimens were procured immediately after surgical resection. A portion of the tumor tissue was placed into sterile 1.2 ml cryo-vials, snap-frozen in liquid nitrogen and stored at -80°C. In addition, a portion of the specimen was placed in a sterile tube containing 10 ml of ice cold RPMI, and transported to the lab within one hour after excision. Thyroid tissues were placed into 100-mm plates containing ice-cold PBS and mechanically dissected and enzymatically (0.2% type IV collagenase/hyaluronidase) digested. Dissociated tissues were filtered through a 150-mm-pore-size nylon mesh to generate a single-cell suspension. Cell suspensions were adjusted to 5x105 cells/mL and processed for Gp78/AMFR surface expression and AMF-FITC uptake by flow cytometry.

Isolation of spheroidal cultures from human thyroid tissue

Fresh PTC, goiter and collateral tissue specimens were subjected to enzymatic digestion (collagenase/hyaluronidase) and supernatants, containing the majority of differentiated cells (e.g. mostly thyrocytes, and some endothelial cells) were removed. Remaining tissue fragments were plated in medium containing 2% FBS and primary "spheroid-like" cell aggregates collected 5-7 days post culture, transferred to separate wells (12-well plates) containing fresh media (2% FBS) and mechanically dissociated into single cell suspensions to generate “secondary cultures”. Primary aggregates of cells with “spheroid-like” structures were observed 5-7 days post culture and imaged after 14-16 days. Secondary cultures were cloned by limiting dilution. Primary fibroblasts were isolated from normal adult resected connective tissues and maintained in culture for the duration of the experiment.

Western blotting

Cell lysates were prepared as previously described and centrifuged for 15 min at 13,000 rpm at 4°C [10]. Tissue lysates were prepared using PARIS-kit (#AM1921, Ambion, Thermofisher Scientific, Canada) as described in the supplied protocol. Approximately 40 mg of total protein lysate was prepared using 6x loading buffer, boiled for 5 min, centrifuged and separated on 10% SDS-PAGE gels, electroblotted onto nitrocellulose membranes, and revealed with indicated primary antibodies, HRP-conjugated secondary antibodies and chemiluminescence. Band intensity was quantified by densitometry relative to β-actin.

Flow cytometry

Flow cytometry of cell surface Gp78/AMFR expression using the anti-Gp78/AMFR monoclonal antibody, 3F3A, and AMF-FITC internalization were performed as previously described [10]. For uptake studies, cells were incubated with 25 µg/ml AMF-FITC, for 30 min at 37°C. Cell surface-bound conjugate was removed with pronase (400 µg/ml) for 5 min. For flow cytometry, at least 50,000 cells were acquired and analyzed using FACSCalibur and FlowJo software (BD Biosciences).

RT-PCR

Clones derived from spheroidal aggregates obtained from PTC, goiter and collateral tissue specimens were lysed using the PARIS-kit for the extraction of total RNA and proteins following the manufacturer protocol. RT-PCR was performed using total RNA, Superscript III reverse transcriptase (Life Technologies, Thermo Fisher Scientific, Canada) and Oligo (dT)-20mer as described previously [10]. Briefly, 1 mg of total RNA, 100 mM Oligo (dT)-20mer and 1 ml of 10 mM dNTPs were added in RNase/DNase free PCR water in 13 ml of final volume. The reaction mixture was heated at 65°C for five minutes and placed two minutes on ice for denaturing. Then 4 ml of 5X reaction buffer, 1 ml of 0.1 M DTT, 1 ml of RNase out (40 U) and 1 ml of Superscript-III (200 U) were added to the reaction mixture and subjected to a RT-PCR reaction as follows: 50°C for 1 hour followed by 70°C for 15 min and then placed on ice. Table 1 shows the set of primers used for PCR amplification and analysis, melting temperature and base pair size of each product. Approximately 2 ml of the total cDNA from RT was used for PCR amplification for each sample and marker gene. PCR master mix, containing 1.5 ml MgCl2, 5 ml 10x PCR buffer, 1 ml of 10 mM dNTPs, 10 mM each, forward and reverse primers (Table 1), 0.3 ml Platinum Taq polymerase was mixed with 2 ml of total cDNA in 50 ml final volume. PCR was performed as follows: 94°C- 2 min, (94°C- 10 sec, gene specific °C Tm, - 15 sec, 72°C- 15-30 sec) x25 cycle, 72°C- 8 min, 4°C hold (Table 1). PCR products were analyzed on 1.5% agarose gel, photo documented and normalized against GAPDH.

**Table 1:** Set of primers used in the study: primer sequence, size of the PCR products and Tm (°C) used for amplification ABCG2: ATP-binding cassette super-family G member 2; Bp: base pairs; (dT)20-mer: oligo(dt)20 primer; GAPDH: glyceraldehyde 3-phosphate dehydrogenase; hGP78/hAMFR: human glycoprotein 78/human autocrine motility factor receptor; OCT-4: octamer-binding transcription factor 4; PAX8: paired box gene 8; PCR: polymerase chain reaction; TG: thyroglobulin; Tm: primer melting temperature; TSHr: thyroid stimulating hormone receptor.

Gene	Forward (5'-3')	Reverse (5'-3')	Size (bp)	Tm (°C)
(dT)20-mer	TTTTTTTTTTTTTTTTTTTT	N/A	N/A	N/A
ABCG2	AGTTCCATGGCACTGGCCATA	TCAGGTAGGCAATTGTGAGG	379	53
OCT-4	GACAACAATGAGAACCTTCAGGAG	CTGGCGCCGGTTACAGAACCA	216	55
PAX8	TCCACCCCTTCCTCTTTATCT	AGTCCTCCTGTTGCTCAGTCG	441	58
TG	AGTCCTAAGTCCCCTGATGC	CAAGGGAGACGTCGAGTGT	280	55
TSHr	AATCCCTGTGAATGCTTTTC	ACTCAAGGAAAGTGGAAGTT	310	55
GAPDH	GGTCGGAGTCAACGGATTTGGTCG	CCTCCGACGCCTGCTTCACCAC	780	58
hGP78/hAMFR	GACCTCCAGCTGACACGCTCAGTTGA	GCTCTCTGAGGCCGCATCATCTTCAG	396	58

Statistical analysis

For TMA analysis the significance of Gp78/AMFR marker expression in DTC, ATC, and benign thyroid lesions, as well as the correlation of clinicopathologic characteristics with the expression of this marker in DTC were assessed with the Pearson χ2 or Fisher’s exact test, where appropriate, for categorical variables and Mann-Whitney U test for continuous variables. Gp78/AMFR marker staining pattern for a set of patient matched lymph node metastases were analyzed for correlation with their corresponding primary cancer specimens by Spearman correlation and for significant differences by marginal homogeneity (MH) test. Statistical Package for the Social Sciences (SPSS) statistical software version 16.0 (SPSS, Chicago, IL) and scripts written in the R programming language (version 2.8.1, R Development Core Team, R Foundation for Statistical Computing, Vienna, Austria) were used for these analyses. P values were corrected for multiple testing using the Benjamini-Hochberg (BH) correction [15]. All statistical tests were two-tailed and a P value of < 0.05 was considered statistically significant.

Unless otherwise stated, all values are presented as mean ± SEM. Statistical significance was evaluated using the Student t-test for paired comparison; P<0.05 was considered significant.

## Results

Tissue microarray analysis of Gp78/AMFR in human thyroid tumors and clinicopathological correlations

**Figure 1:**
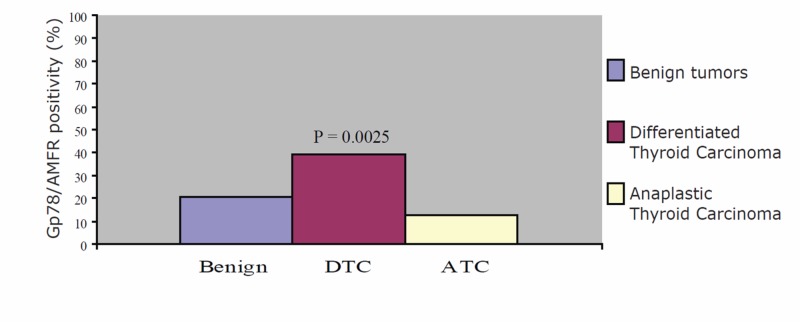
Tissue microarray analysis of human thyroid carcinomas and lymph nodes Graph showing % expression of Gp78 AMFR in benign, DTC and ATC specimens. ATC: anaplastic thyroid carcinoma; DTC: differentiated thyroid carcinoma; Gp78/ AMFR: glycoprotein 78 /autocrine motility factor receptor.

DTC displayed more immunoreactivity than did the benign lesions and the ATCs (39.1% vs. 20.4% and 12.5%, respectively; P = 0.0025). Gp78/AMFR showed significantly elevated labeling of DTC relative to either benign or ATC as can be seen in Figure 1 and Table 2. The utility of Gp78/AMFR for diagnostic purposes has an accuracy, sensitivity, specificity and precision of 58.2%, 39.1%, 79.6% and 68.3%, respectively. The expression of Gp78/AMFR was also assessed in primary PTCs and their associated lymph node metastases, where present (Table 3). Two lymph node specimens, and their corresponding associated primary PTC, were excluded because insufficient cancer metastasis tissue was available for evaluation. The Spearman correlation test suggested that the primary PTCs and associated lymph node metastases had Gp78/AMFR expression that was slightly correlated; however, the correlation was not statistically significant (ρ = 0.381, correlation P value = 0.131). The MH test suggested that there was no significant difference (MH P value = 0.180) in expression of Gp78/AMFR between the primary PTCs and their matched lymph node metastases (Table 4).

**Table 2:** Marker expression in benign, DTC and ATC specimens ATC: anaplastic thyroid carcinoma; DTC: differentiated thyroid carcinoma; Gp78/ AMFR: glycoprotein 78 /autocrine motility factor receptor.

Marker	% positive benign specimens	% positive DTC specimens	% positive ATC specimens	P value
Gp78/AMFR	20.4	39.1	12.5	0.0025

**Table 3:** Gp78/AMFR marker expression in DTC primary tumors and associated lymph node metastases DTC: differentiated thyroid carcinoma; Gp78/ AMFR: glycoprotein 78 /autocrine motility factor receptor; LN: lymph node.

Gp78/AMFR expression	Primary+ LN+	Primary+ LN+	Primary+ LN+	Primary+ LN+
Cases (%)	3 (17.6)	1 (5.9)	4 (23.5)	9 (52.9)

**Table 4:** Percent of DTC primary tumors and their lymph node metastases expressing Gp78/AMFR DTC: differentiated thyroid carcinmoa; Gp78/ AMFR: glycoprotein 78 /autocrine motility factor receptor; NS: not significant.

Marker	% positive benign specimens	% positive DTC specimens	P value
Gp78/AMFR	23.8	41.2	NS

Differentiated thyroid cancer cell lines abundantly express Gp78/AMFR and readily internalize AMF

**Figure 2:**
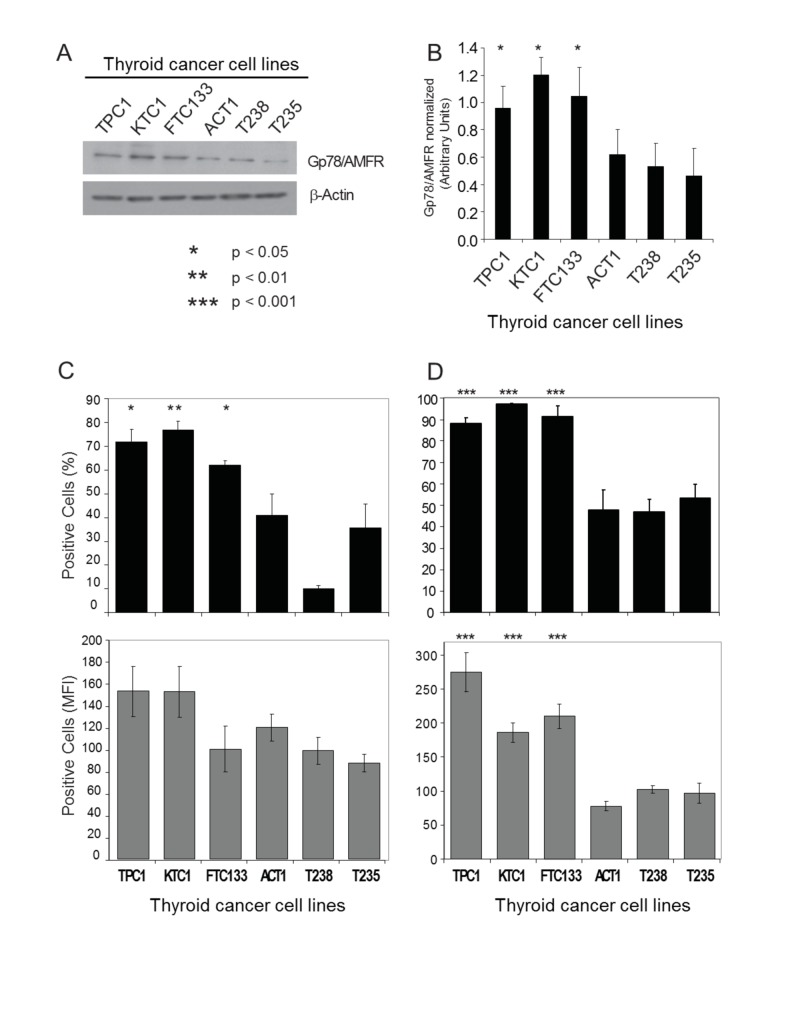
Gp78/AMFR expression and AMF-FITC internalization in human thyroid cancer cells Six thyroid cancer cell lines (PTC: TPC1, KTC1; FTC: FTC133; ATC: ACT1; T238; T235) were profiled for Gp78/AMFR expression and AMF-FITC internalization. (A) Cell lysates were analyzed for total Gp78/AMFR protein expression by Western blot as shown in a representative blot. (B) The graph shows the densitometric quantification of Western blot bands in (A) across four separate experiments (mean ± SEM). To determine cell surface expression of Gp78/AMFR, cells were detached by non-enzymatic treatment with EDTA at 37 °C, stained with anti-Gp78/AMFR monoclonal antibody followed by Alexa-647 conjugated secondary antibody at 4 °C, and analyzed by FACS. Gp78/AMFR surface expression is shown in (C) as relative quantitative analysis of the percentage of positive cells (top graph) and changes in MFI (bottom graph) are shown. To determine AMF internalization, human thyroid cancer cells were incubated with 25 µg/ml of AMF-FITC for 30 min at 37 °C, treated with pronase (400 µg/mL) for 5 min at 37 °C and processed for FACS analysis. AMF-FITC internalization is shown in (D) as relative quantitative analysis of the percentage of positive cells (top graph) and changes in MFI (bottom graph) are shown. The data represent the average of at least three separate experiments (mean ± SEM; * P<0.05; ** P< 0.01; *** P<0.001, relative to ATC cells). AMF: autocrine motility factor; AMF-FITC: autocrine motility factor- fluorescein isothiocyanate; ATC: anaplastic thyroid cancer; EDTA: ethylenediaminetetraacetic acid; FACS: fluorescence-activated cell sorting; FITC: fluorescein isothiocyanate; Gp78/AMFR: glycoprotein 78 /autocrine motility factor receptor; MFI: mean fluorescence intensity; PTC: papillary thyroid cancer; SEM: standard error mean.

We profiled six human thyroid cancer cell lines: two papillary (TPC1, KTC1), one follicular (FTC133), and three anaplastic (ACT1, T238, T235). These thyroid cell lines have been genetically profiled and their authenticity validated [16]. First, we assessed the total expression of Gp78/AMFR in the thyroid cancer cell lines. Consistent with the TMA data, immunoblot of total cellular protein with the 3F3A anti-Gp78/AMFR monoclonal antibody showed approximately two-fold increased expression of Gp78/AMFR in the three DTC cell lines relative to the anaplastic cell lines (Figure 2A). We next determined the surface expression of Gp78/AMFR receptor on the thyroid cancer cell lines by flow cytometry with the 3F3A antibody. Cell surface Gp78/AMFR was elevated for the three DTC relative to the ATC cell lines with the T238 ATC cell line showing significantly reduced cell surface Gp78/AMFR expression (Figure 2B).

To study AMF internalization, we incubated the cells with FITC-conjugated AMF for 30 minutes at 37^o^C and pronase-treated the cells to remove cell surface bound AMF-FITC [10]. By FACS analysis, AMF-FITC internalization was significantly increased in human PTC and FTC compared to ATC cell lines (Figures 2B). This was observed with respect to both cell positivity and mean fluorescent intensity, suggesting that PTC and FTC cell lines that expressed high levels of Gp78/AMFR have increased binding and intracellular uptake of the AMF-FITC conjugate. Reduced Gp78/AMFR expression in the ATC cell lines was associated with significantly reduced (2-3 fold lower; P<0.001) internalization of AMF-FITC.

Gp78/AMFR surface expression and AMF-FITC internalization in human thyroid cancer

**Figure 3:**
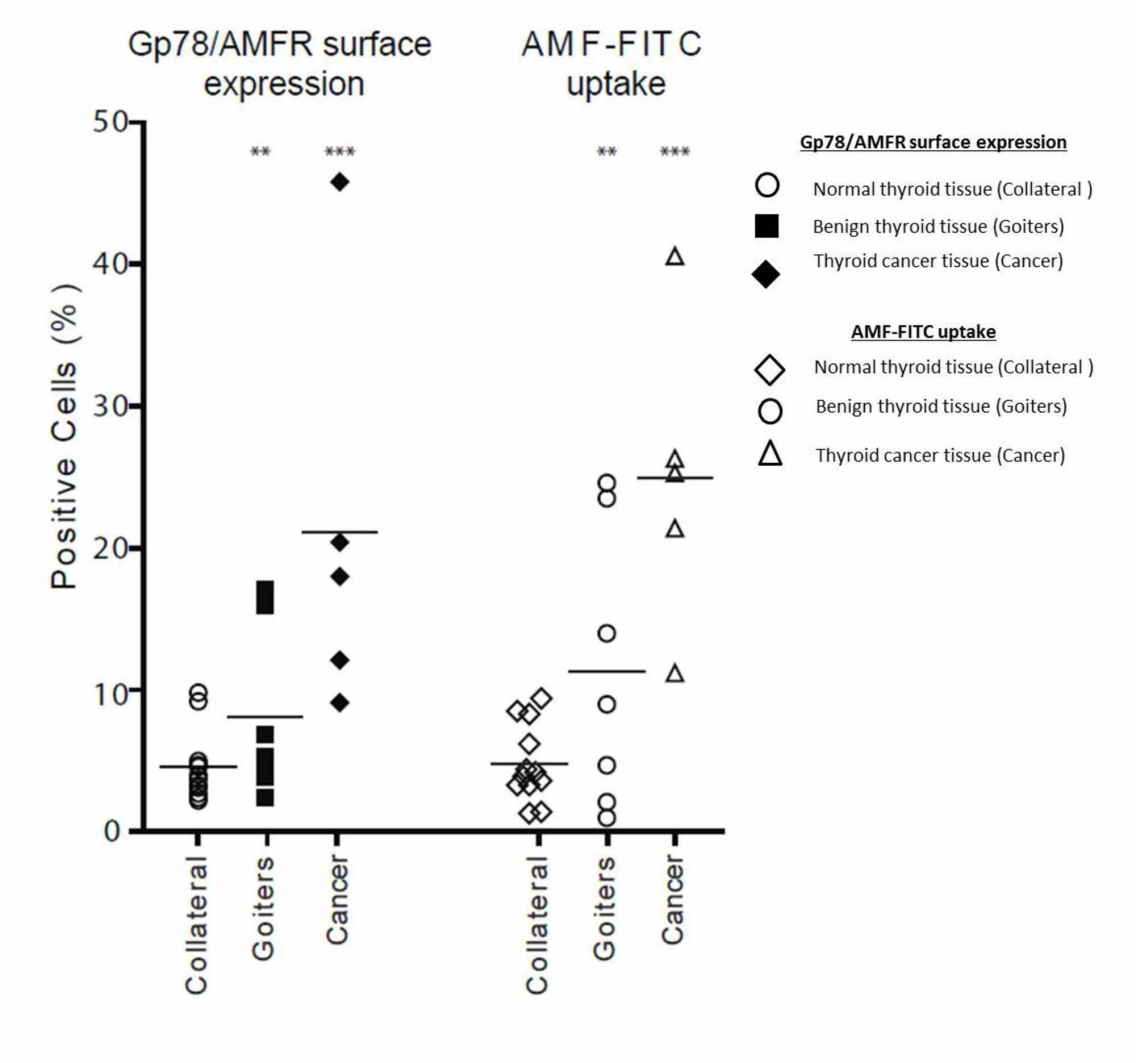
Gp78/AMFR surface expression and AMF internalization in malignant, benign and normal thyroid tissues Cell surface Gp78/AMFR expression and AMF-FITC uptake in isolated cells from freshly resected human thyroid cancer, benign and normal tissues were assessed by flow cytometry. The percentage of positive cells is presented. The data represent the average of twenty separate experiments (mean ± SEM; **, P < 0.01; ***, P < 0.001). AMF: autocrine motility factor; AMF-FITC: autocrine motility factor- fluorescein isothiocyanate; Gp78/AMFR: glycoprotein 78 /autocrine motility factor receptor; SEM: standard error mean.

To determine whether human thyroid cancers can internalize AMF, we assessed Gp78/AMFR surface expression and AMF-FITC uptake in freshly resected primary human thyroid tissues, including five pathologically confirmed PTCs, seven benign thyroid lesions, and 12 normal collateral thyroid tissue specimens. The freshly isolated tissues were mechanically and enzymatically dissociated before analysis by flow cytometry for cell surface Gp78/AMFR expression and AMF-FITC uptake. All PTCs were Gp78/AMFR-positive and demonstrated significantly higher Gp78/AMFR surface expression (P<0.001) compared to non-cancerous thyroid tissues (Figure 3). Furthermore, Gp78/AMFR-positive thyroid cancers showed significantly increased (P<0.001) internalization of AMF-FITC compared to non-malignant tissues. Gp78/AMFR expression and AMF internalization were reduced but still detected in cells isolated from both benign tumors and normal collateral thyroid tissue. This suggests that a sub-population of cells that express Gp78/AMFR and internalize AMF is enriched in the cancers.

Gp78/AMFR expression and AMF internalization in potential thyroid cancer stem cells 

**Figure 4:**
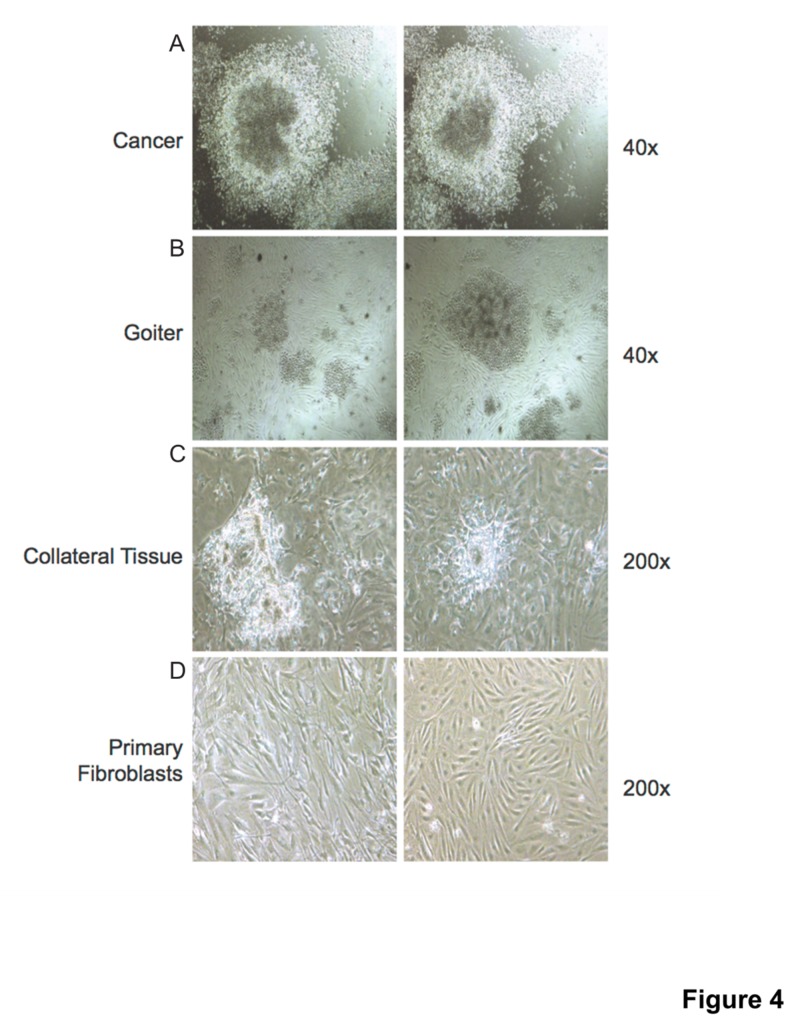
Isolation of spheroid-like colonies from thyroid tissue Fresh human thyroid tissues (cancer, goiter and collateral) were collected and subjected to enzymatic digestion (collagenase/ hyaluronidase). Thyroid tissue explants were cultured in medium containing 2% FBS and primary aggregates of cells with "spheroid-like” structures were observed 5-7 days post culture. "Spheroid-like” aggregates were imaged after 14-16 days post culture and two representative images are shown for (A) thyroid cancer explants at 40x magnification, (B) goiter explants at 40x magnification, and (C) collateral tissue explants at 200x magnification. (D) Primary fibroblasts were isolated from normal adult resected connective tissues and maintained in culture for the duration of the experiment and are shown as two representative figures at 200x magnification. FBS: fetal bovine serum.

Freshly resected PTC, benign thyroid lesions, and collateral normal thyroid tissue specimens were subjected to enzymatic digestion (collagenase/hyaluronidase) and tissue fragments were plated in medium containing 2% FBS. Primary "spheroid-like" cell aggregates were collected 5-7 days post-culture and mechanically dissociated into single cell suspensions to generate “secondary cultures”. Primary aggregates of cells with “spheroid-like” structures were observed 5-7 days post-culture and imaged after 14-16 days (Figure 4). Primary fibroblasts were isolated from normal adult resected connective tissues and maintained in culture for the duration of the experiment.

**Figure 5:**
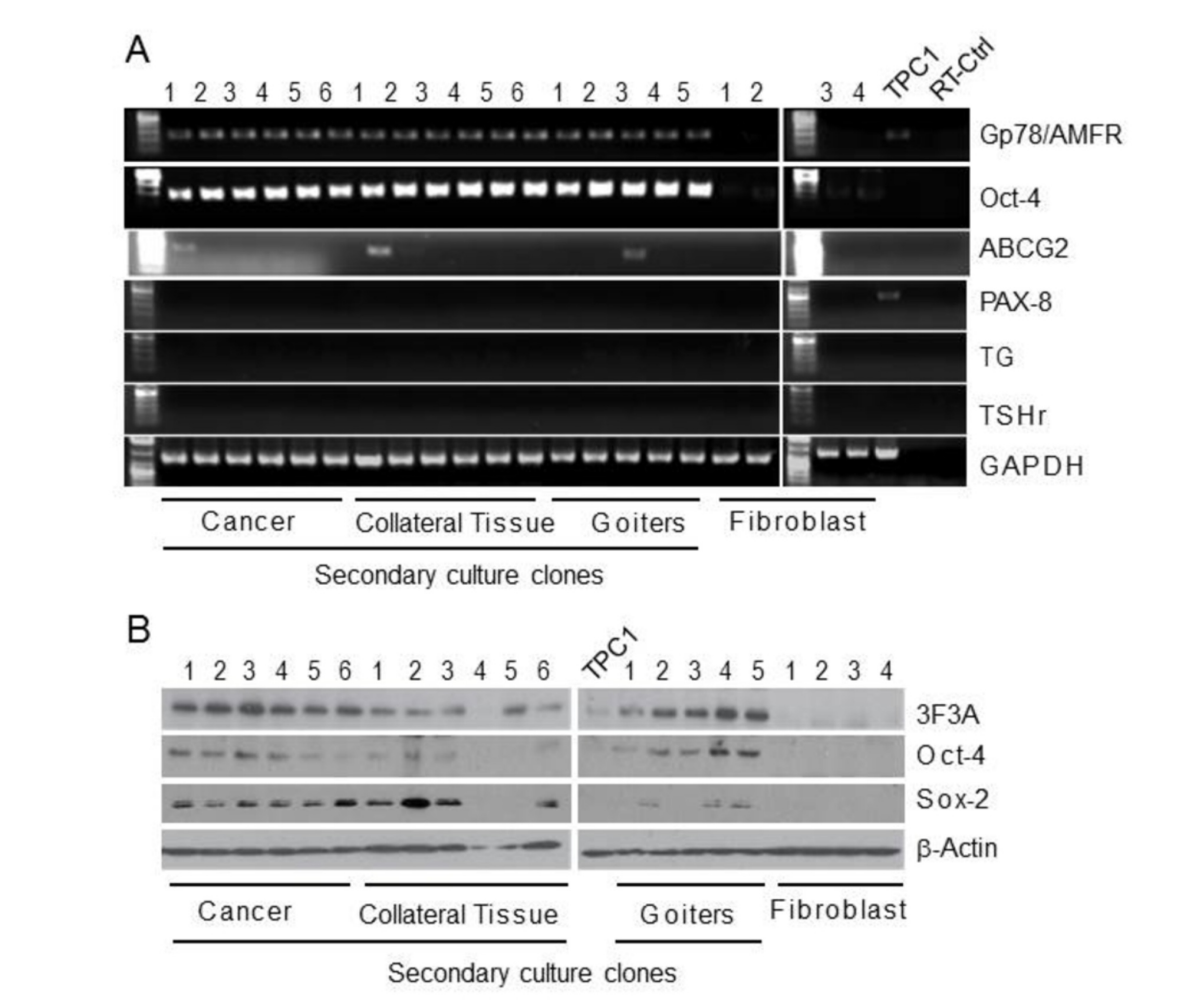
Profiling clones derived from spheroid-like thyroid cultures (A) Total RNA of clones derived from spheroid-like cultures of cancer, goiter and collateral thyroid tissue, primary fibroblasts and the Gp78/AMFR-expressing TPC1 DTC cell line (as a control) were analyzed by RT-PCR for the expression of mRNA for the Oct-4 and ABCG2 stem cell markers, the PAX-8, TG and TSHr differentiation markers, and Gp78. GAPDH mRNA expression was used as a housekeeping gene transcript control. (B) Cell lysates of clones from spheroid-like cultures of cancer, goiter and collateral tissue, primary fibroblasts and the Gp78/AMFR-expressing TPC1 DTC cell line were analyzed by Western blot for expression of total Gp78/AMFR (3F3A mAbs) and the stem cell markers Oct-4 and Sox-2 followed by HRP-conjugated secondary antibodies. β-actin was used as a loading control. ABCG2: ATP-binding cassette super-family G member 2; DTC: differentiated thyroid cancer; GAPDH: glyceraldehyde 3-phosphate dehydrogenase; Gp78: glycoprotein 78; Gp78/AMFR: glycoprotein 78 /autocrine motility factor receptor; HRP: horseradish peroxidase; Oct-4: octamer-binding transcription factor 4; PAX-8: paired box gene 8; Sox-2: sex determining region Y)-box 2; TG: thyroglobulin; TSHr: thyroid stimulating hormone receptor.

Colonies were cloned from the secondary cultures and analyzed by RT-PCR for mRNA expression of Gp78/AMFR, the tumor stem cell gene markers ABCG2 and Oct-4 and the thyroid differentiation markers thyroglobulin (TG) and thyroid stimulating hormone receptor (TsHR) (Figure 5A). All colonies but not fibroblasts were found to express Gp78/AMFR and Oct-4 mRNA. Select individual clones of the colonies from PTC, benign thyroid lesions and collateral normal thyroid tissue were also found to express ABCG2. PAX-8, TG and TsHR were not detected in any of the clones, including fibroblasts. The thyroid cancer TPC1 cell line expressed Gp78/AMFR and PAX-8 mRNA. By Western blot, all cancer-derived colonies and most of those derived from benign thyroid lesions or collateral normal thyroid tissue expressed Oct-4 and Sox-2 protein (Figure 5B).

**Figure 6:**
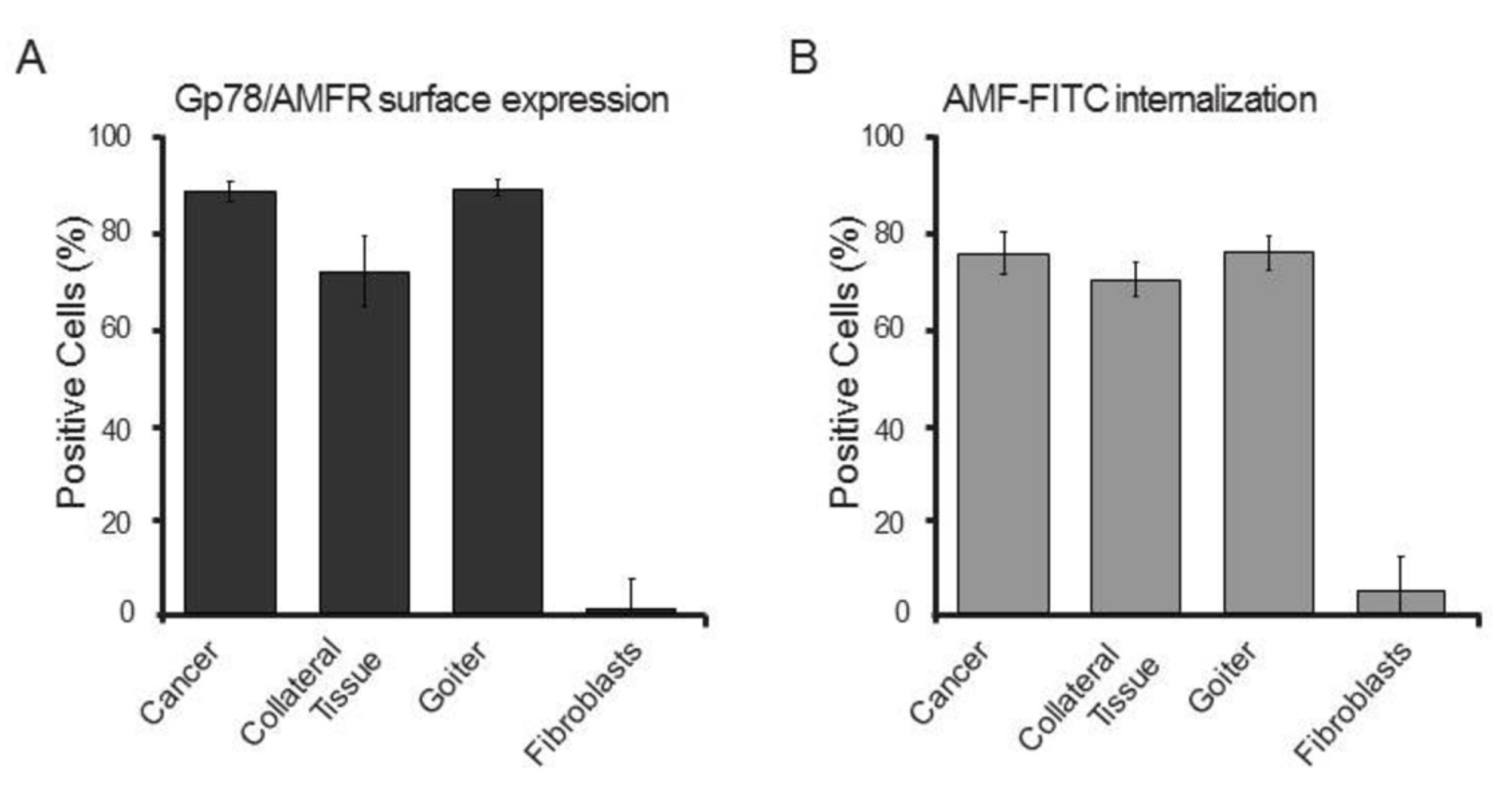
Gp78/AMFR surface expression and AMF internalization in clones derived from spheroid-like thyroid cultures Clones from spheroid-like cultures of cancer, goiter and collateral tissue as well as fibroblasts isolated from these cultures were profiled by FACS for (A) Gp78/AMFR cell surface expression and (B) AMF-FITC endocytosis. AMF: autocrine motility factor; AMF-FITC: autocrine motility factor- fluorescein isothiocyanate; FACS: fluorescence-activated cell sorting; Gp78/AMFR: glycoprotein 78/autocrine motility factor receptor.

By Western blot, PTC, goiter and collateral tissue derived clones were found to express Gp78 (Figures 5B). Gp78/AMFR expression was not detected in fibroblasts. Oct-4 was detected in both tumor and goiter clones. FACS analysis showed that clones from cancers and benign thyroid lesions express high levels of surface Gp78/AMFR and efficiently internalize AMF-FITC (Figure 6), compared with primary adult human fibroblasts. These observations suggest that compared to normal fibroblasts, Gp78/AMFR expression and AMF internalization are elevated in a subpopulation of thyroid cancer cells that exhibit cancer stem cell (CSC) growth characteristics and express CSC markers.

## Discussion

The current study is the first to comprehensively evaluate Gp78/AMFR expression and AMF internalization in thyroid cancer. A multifaceted analysis of thyroid TMAs, human thyroid cancer cell lines and primary human thyroid cancers has shown that Gp78/AMFR expression and AMF uptake are more closely associated with DTC compared to benign thyroid lesions or ATC. The subpopulation of cells positive for Gp78/AMFR surface expression and AMF internalization increases progressively in collateral thyroid tissue, benign thyroid cancers and papillary thyroid cancers with 20% of the PTC cells positive. This is consistent with increased Gp78 expression in DTC by TMA analysis.

Thyroid cancer stem cells have been identified and isolated from human thyroid glands based upon their higher aldehyde dehydrogenase (ALDH) activity [17], expression of ATP-binding cassette super-family G member 2 (ABCG2) [18, 19] and CD133 [20]. The propagation of highly proliferating stem cells in response to acute intense growth stimulation resembles the chronic growth stimulation present in nodular goiters, which may suggest a pathogenetic role of stem cells in goiterogenesis [19]. Spheroidal aggregates isolated from PTCs express CSC markers and are potential thyroid CSCs. They show elevated expression of Gp78/AMFR and robust internalization of AMF. However, the 20% of PTC cells that express Gp78/AMFR and internalize AMF contrasts the 1-2% of PTCs that are clonogenic and form spheres [17]. Further, the reduced Gp78/AMFR expression observed in ATC by TMA contrasts the increased abundance of CSCs reported in ATC [17]. Expression of CSC markers by the clonal aggregate-derived cells that show elevated Gp78/AMFR expression and AMF internalization is suggestive of an association of Gp78/AMFR-dependent AMF internalization in CSCs. However, further studies with better characterized CSCs are required to determine whether Gp78/AMFR and AMF are CSC biomarkers.

Gp78/AMFR expression is closely associated with tumor metastasis [13, 21, 22] and with a poor prognosis in several cancer types (reviewed in [6]). Metastatic cancer cells exhibit experimental and clinical behaviors highly reminiscent of the classical properties of stem cells, including resistance to current chemotherapy regimens [23]. AMF has been shown to exhibit chemoprotective ability, protecting tumor cells from the cytotoxic effects of various chemotherapeutic agents including paclitaxel [11, 24]. However, Gp78/AMFR was recently reported to be a tumor suppressor in liver [8]. TMA analysis in the same study of colon and rectal cancers showed that Gp78/AMFR expression was associated with improved or worsened prognosis, respectively, in these two closely related but distinct cancer types [9]. Importantly, these studies as well as those described here were performed with the 3F3A antibody that does not recognize p38 MAP kinase phosphorylated (on serine 538) Gp78 [7] such that TMA analysis with the 3F3A mAb is not reporting on the totality of Gp78 expression. The role of p38 MAP kinase and Gp78 phosphorylation on Gp78 function in cancer progression is not known. Indeed, how Gp78/AMFR can function to both promote and suppress cancer progression is poorly understood. 

A further explanation for this observation may be the dual function of Gp78/AMFR as cell surface AMF receptor and key ER-localized ubiquitin ligase in ERAD. Gp78/AMFR knockout mice show elevated ER stress levels in liver, consistent with the key role of Gp78/AMFR ubiquitin ligase activity in ERAD [8, 25]. At the same time, AMF protects cells against ER stress and associated cell death [26]. Gp78/AMFR also increases ER-mitochondria association, degrading the mitofusin mitochondrial fusion proteins and inducing mitochondrial fission and mitophagy [27, 28]. AMF reverses Gp78/AMFR promotion of ER-mitochondria interaction and induces mitochondrial fusion [28]. ER-mitochondria association is closely associated with apoptosis and cell death [29]. AMF reversal of Gp78/AMFR-dependent ER-mitochondria interaction and protection against ER stress induced cell death [26, 28] may thereby promote tumor cell survival under conditions of elevated ER stress that are closely associated with cancer progression [30].

## Conclusions

Gp78/AMFR expression and AMF uptake are more closely associated with DTC compared to benign thyroid lesions or ATC and with PTC-derived cancer stem-like cells. Further study of Gp78/AMFR and AMF uptake by thyroid cancer is warranted and may allow for the development of novel diagnostic and prognostic tools, and therapies, for this common human endocrine cancer type.
